# Pyrrolopyrrole-Based Aza-BODIPY Small Molecules for Organic Field-Effect Transistors

**DOI:** 10.3389/fchem.2022.938353

**Published:** 2022-06-27

**Authors:** Daohai Zhang, Dongxu Liang, Liang Gu, Haichang Zhang

**Affiliations:** ^1^ School of Chemical Engineering of Guizhou Minzu University, Guiyang, China; ^2^ Key Laboratory of Rubber-Plastics of Ministry of Education/Shandong Province (QUST), School of Polymer Science and Engineering, Qingdao University of Science and Technology, Qingdao, China

**Keywords:** pyrrolopyrrole-based aza-BODIPY, electron-deficient, conjugated materials, hole transport mobility, organic field-effect transistor

## Abstract

Diketopyrrolopyrrole (DPP), due to its good planarity, π-conjugate structure, thermal stability, and structural modifiability, has received much attention from the scientific community as an excellent semiconductor material for its applications in the field of optoelectronics, such as organic solar cells, organic photovoltaics, and organic field effect transistors. In this study, a new small molecule, pyrrolopyrrole aza-BODIPY (PPAB), based on the thiophene-substituted DPP structure was developed using the Schiff-base formation reaction of DPP and heteroaromatic amines. Absorption spectroscopy, electrochemistry, X-ray diffraction, molecular theoretical simulation calculation were performed, and organic field-effect transistor properties based on PPAB were investigated. It was found that PPAB exhibits a broad absorption range in the visible and near-infrared regions, which is attributed to its long-range conjugate structure. In addition, it is worth noting that PPAB has multiple F atoms resulting in the low LUMO level, which is conducive to the injection and transportation of charge carriers between the semiconductor layer and the electrode. Meanwhile, its hole carrier mobility is up to 1.3 × 10^−3^ cm^2^ V^−1^ s^−1^ due to its large conjugate structure, good intramolecular charge transfer effect, and high degree of coplanarity. In this study, a new chromophore with electron-deficient ability for designing high-performance semiconductors was successfully synthesized.

## Introduction

Diketopyrrolopyrrole (DPP), ever since it was first discovered in the 1970s, has great potential as an excellent semiconductor material for optoelectronic devices (Yao et al., 2015; Mitchell and Jones, 2018) because of its excellent molecular structural characteristics such as excellent planarity, large conjugated molecular skeleton, structural modifiability, and good stability, which has attracted extensive research interest (Rochat et al., 1985; Guo et al., 2014; Zhao et al., 2016; Huang et al., 2017; Zhang et al., 2017). Recently, in-depth research was conducted on the DPP structure and hundreds of DPP derivatives were reported. These DPP derivatives show excellent performance in various electronic devices because of their unique molecular core structure, which greatly promotes the development of organic electronic device materials (Luo et al., 2016; Oh et al., 2016).

The organic field-effect transistor (OFET) is a kind of device that relies on the external electric field to induce current on the surface of the semiconductor layer, which can be changed by regulating the intensity of the external enhanced electric field (Zhang et al., 2020). Because of its small size, light weight, low power consumption, and special variable conductivity, OFET has become one of the most important components in the microelectronics industry. Charge carrier mobility is one of the most critical parameters used to characterize the device performance, which directly reflects the carrier movement in the semiconductor material layer (Zhao et al., 2016; Shi et al., 2020). To obtain OFETs with high performance and high electron mobility, the designed semiconductor layer molecules generally need to have a longer conjugate structure, good planarity and π–π stacking structure, and an excellent intramolecular charge transfer (ICT) effect, which is beneficial to match the energy level of the metal electrode. Therefore, the DPP structure is one of the best candidate structures (Qu and Tian, 2012; Yuto et al., 2018). In addition, in order to ensure a lower LUMO level, it is necessary to introduce atoms or groups with greater polarity, such as nitrogen atoms, fluorine atoms, and cyano groups (Jo et al., 2015). These groups can be introduced in the process of structural modification of DPP. At the same time, they can also give it better planarity and conjugate structure. Although molecules with high carrier mobility have been developed recently, the continuous development of new strategies for high-carrier-mobility molecules is still an important topic.

**FIGURE 1:**
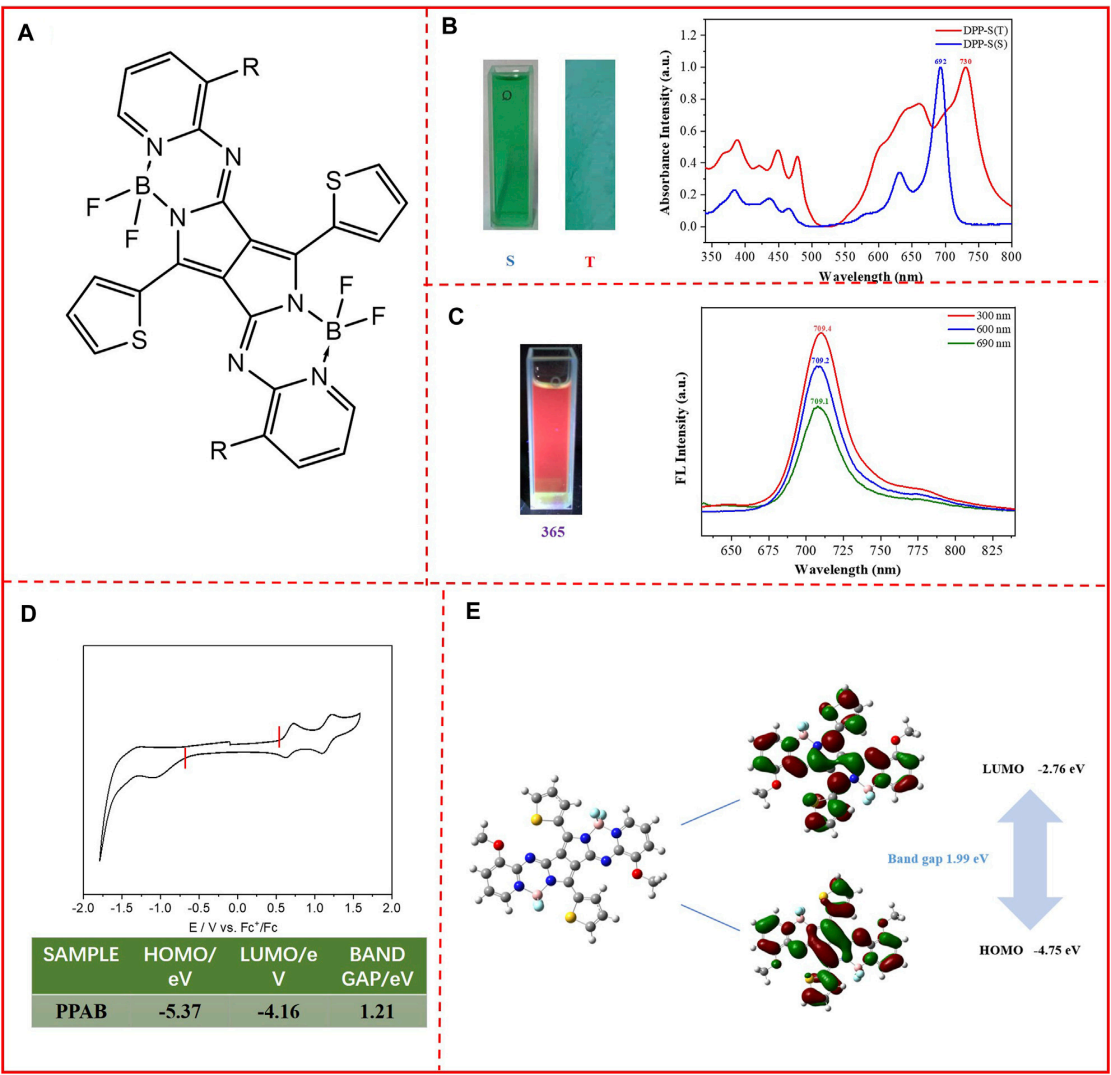
**(A)** Chemical structure of PPAB; **(B)** photos of PPAB in the chloroform solution (left) and in the thin film state (right). UV/vis absorption spectra of PPAB in the chloroform solution (S) and thin films (T); **(C)** photos of PPAB in the chloroform solution excited by 365 nm light; PL spectra of PPAB in chloroform, excited by 300, 600, and 690 nm; **(D)** cyclic voltammograms of PPAB. Electrolyte: 0.1 M TBAPF_6_ in acetonitrile. Potential calculated vs. ferrocene. Scan rate: 100 mV s^−1^; *T* = 25°C; **(E)** computational calculations of the simplified PPAB obtained at the B3LYP/6-31G* level and molecular orbital surfaces of the HOMO and LUMO energy levels and the band gap of PPAB.

In this study, a new small molecule (pyrrolopyrrole aza-BODIPY, or PPAB; Figure 1A) based on the DPP structure was synthesized using the Schiff-base formation reaction between thiophene-substituted DPP and heteroaromatic amines, and in this structure, four fluorine atoms and a large number of N atoms are also introduced, which are in favor of reducing the LUMO level (Donaghey et al., 2013; Wiktorowski et al., 2014; Lin et al., 2018; Lee et al., 2019). The 3,6 positions of the DPP structure were replaced with thiophene groups to improve the electron enrichment of molecules (Sonar et al., 2012; Murlidhar, 2018; Li et al., 2014; Jiang et al., 2017; Li et al., 2017). More importantly, the extension was performed using the B―N bridge in the longitudinal direction of the structure to improve the π–π stacking area (Dou et al., 2016; Long et al., 2016). The results indicate that PPAB shows a lower LUMO level of about −2.76 eV. OFET devices were also prepared with PPAB as the semiconductor layer, and the carrier mobility measured was up to 1.3 × 10^−3^ cm^2^ V^−1^ s^−1^, which is mainly due to the synergy between its large conjugate structure that is conducive to carrier transport and good planarity that is beneficial to improving the π–π stacking area (Yuto et al., 2018; Bao et al., 2020). This study also has great potential not only in designing small molecules but also in the block construction of polymers.

## Results and Discussion

### Optical Properties

The molecule presents a green color in both the chloroform solution and film state (Figure 1B). In order to investigate the optical properties of PPAB, the absorption spectra of PPAB in the thin film state and in the chloroform solution were measured. It was found that PPAB exhibits a broad absorption range between 550 and 800 nm. In the chloroform solution, the maximum absorption peak occurs at 692 nm, while in the solid film, the maximum absorption peak shifts to 730 nm. This red shift of nearly 40 nm can be attributed to the aggregation and tight packing of molecules in the solid film compared to the solution, and PPAB tends to exist in the form of single molecules in the solution. From the onset optical absorption, the optical band gap is calculated to be 1.55 eV. In addition, the chloroform solution of PPAB can exhibit an obvious red color with the excitation of 365 nm light as shown in Figure 1C. The photoluminescence (PL) spectra were also recorded; due to its wide range of absorption, three different wavelengths of light (300, 600, and 690 nm) were chosen to excite it. The results are shown in Figure 1C; the emission peaks of PPAB are observed at 709 nm, with a red shift of nearly 20 nm relative to 692 nm corresponding to the maximum absorption peak. The experiment of fluorescence emission was also carried out in the thin film, but it did not show fluorescence emission, which might be caused by the aggregation-induced quenching effect. This indicates the PPAB thin film with a strong aggregation between molecules and the formation of excimer association complexes or excimer complexes by π–π interactions, which consumes the energy of excited states.

### Electrochemical Properties and Molecular Theoretical Simulation

According to the initial reduction potential and oxidation potential, the LUMO/HOMO energy level of the monomer can be estimated. In Figure 1D, the PPAB monomer shows a reversible and redox curve, and the first initial oxidation and reduction positions appear at 0.57 and −0.64 V, respectively. Based on this, the HOMO and LUMO energy levels are −5.37 eV and −4.16 eV, respectively. The energy band gap is about 1.21 eV, which is 0.33 eV smaller compared to the optical band gap. It is worth noting that the four fluorine atoms and a deal of nitrogen atoms introduced play an important role in the reduction of LUMO energy level as the result of increasing the electron-deficient ability of the molecular skeleton, which is in favor of matching with the metal electrode.

In order to further determine the characteristics of the molecular structure, computation calculations were performed by density-functional theory (DFT) at the B3LYP/6-31 (d,p) level using simplified chromophores with the methyl group instead of the alky chain. It was found that the molecular skeleton has good coplanarity, which is conducive to the formation of π–π stacking between adjacent molecules (Figure 1E). Furthermore, the electrons in the HOMO orbitals were mainly localized at the thiophene DPP with part of the core of the backbone, while the LUMO orbitals were mainly localized at the core of PPAB with part of the electron wave function along the conjugation backbone structure, and the HOMO and LUMO orbitals show a relatively even state, which means that the molecule has a potentially strong ICT effect. The PPAB measured by cyclic voltammetry may have a certain degree of molecular aggregation, which leads to a level different from the HOMO and LUMO energy levels calculated by theoretical simulation. The narrow band gap measured by the cyclic voltammetry shows the strong interaction of molecules in the aggregated state, which is conducive to the effective extension of conjugation for carrier transportation to a certain extent.

### X-Ray Diffraction

**FIGURE 2:**
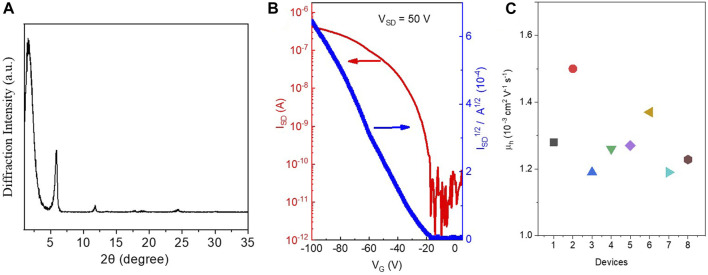
**(A)** Transmission XRD diagrams of PPAB; **(B)** characteristics of the OFET devices of PQ1; **(C)** hole transport mobility obtained from eight different devices.

In order to better understand the structure of this small molecule, X-ray diffraction (XRD) was used to investigate and to characterize the structure of the PPAB thin film (Figure 2A). The XRD patterns of PPAB exhibit primary diffraction peaks at 2*θ* = 1.56°, 5.56°, and 11.78° for PPAB, which correspond to the inter-lamellar distance of 1.935 nm. The peak shape also indicates that the molecule will have a preferential growth trend during aggregation, which is consistent with the flake state of the molecule. In addition, this relatively compact structure is consistent with the results of molecular theoretical simulation mentioned above.

### Organic Field-Effect Transistors

The charge transport properties of PPAB were evaluated by fabricating OFET devices of PPAB in a bottom-gate and bottom-contact configuration on a silicon wafer using a layer of 300 nm SiO_2_ as the dielectric material. The devices with chromophores were fabricated by directly drop-coating the chloroform solution of the compound onto the octadecyltrichlorosilane-treated silicon wafer with pre-patterned gold source and drain electrodes. The devices were measured under the vacuum condition after thermal annealing at 50°C for 5 min in an argon-filled glove box to remove the remaining residual solvent. The PPAB-based device exhibits p-type semiconductor properties with an average hole transport mobility (μ_h_) of around 1.3 × 10^−3^ cm^2^ V^−1^ s^−1^ (the highest μ_h_ of 1.5 × 10^−3^ cm^2^ V^−1^ s^−1^; Figures 2B,C). The high charge carrier mobility of PPAB is mainly ascribed to two factors: 1) PPAB shows a high degree of coplanarity of the molecular core and the large π-conjugation system, which is beneficial for the charge transport within the individual molecules; 2) the PPAB film features strong aggregation, which is an advantage for the charge transport between the adjacent molecules. The I_on_/I_off_ ratio for the IIDG-AB-based OFETs is in the range of 10^5^∼10^6^, and the threshold voltage for the device operation is −19 V.

## Conclusion

In summary, an electron-deficient chromophore, namely, PPAB, was successfully synthesized by a Schiff-base reaction between thiophene-substituted DPP and heteroaromatic amines in the presence of titanium tetrachloride. The PPAB compound shows an ultra-low LUMO level (only around −4.16 eV). The study of optical properties indicates that there is around 40 nm bathochromic shift for PPAB from the solution to the thin film state, which indicates a strong aggregation with a broad optical absorption range between 500 and 800 nm, which makes it a potential light absorber for photovoltaic applications. The OFETs constructed by PPAB as the semiconductor layer present a clear p-type behavior with a maximum electron mobility of 1.5 × 10^−3^ cm^2^ V^−1^ s^−1^. Our results indicate that PPAB is a promising electron-deficient chromophore to construct semiconductors for OFETs. In addition, we believe that this study can pave the way for future molecular design and engineering to fabricate high-performing semiconductor materials.

## Data Availability

The original contributions presented in the study are included in the article/Supplementary Material; further inquiries can be directed to the corresponding authors.
