# Supplementary material for: Pyrrolopyrrole-Based Aza-BODIPY Small Molecules for Organic Field-Effect Transistors
Source: Front Chem. 2022 Jun 27;10:938353. doi: 10.3389/fchem.2022.938353 (PMC9271750; doi:10.3389/fchem.2022.938353)
Supplement: Supplementary file 1 [file Presentation1.pdf]

Supporting information

Pyrrolopyrrole-based Aza-BODIPY (PPAB) Small Molecule for Organic Field-Effect Transistors

Daohai Zhang,[1a*] Dongxu Liang[1b] Liang Gu,[b] Haichang Zhang[b]*

[a] School of Chemical Engineering of Guizhou Minzu University, Guizhou,Guiyang 550025, China

[b]Key Laboratory of Rubber-Plastics of Ministry of Education/Shandong Province (QUST), School of Polymer Science and Engineering, Qingdao University of Science and Technology, 53-Zhengzhou Road, Qingdao 266042, P. R. China

Corresponding author: Daohai Zhang: zhangdaohai6235@163.com; Haichang Zhang: haichangzhang@hotmail.com

Contents

1. Experimental Procedures
1.1 Materials
1.2 Synthesis of PPAB
Synthesis of **(1) 2-Amino-3-(2-hexyldecyloxy)pyridine**
Synthesis of **(2) 3,6-Di(thiophen-2-yl)-2,5-dihydropyrrolo[3,4-c]pyrrole-1,4-dione**
Synthesis of **(3) PPAB**
1.3 Characterization for polymers
1.3.1 Structure and molecular weight characterization
1.3.2 Thin film structures characterization
1.3.3 UV/vis absorption spectra of monomer and polymers
2. Figures
SI-Figure 1. The ^{1}H NMR spectrum of (1) 2-Amino-3-(2-hexyldecyloxy)pyridine.
SI-Figure 2. The ^{1}H NMR spectrum of (3) PPAB.
3. Table
Table S1. The corresponding photoelectric property data of PPAB.
4. Reference

1. Experimental Procedures

1.1 Materials

All reagents were purchased from commercial sources and used without further purification unless otherwise noted. All the solvents needed in the experiment were dried and distilled by metal sodium and then used fresh, and all reaction processes are protected by nitrogen.

1.2 Synthesis route of PPAB

(1) 2-Amino-3-(2-hexyldecyloxy)pyridine

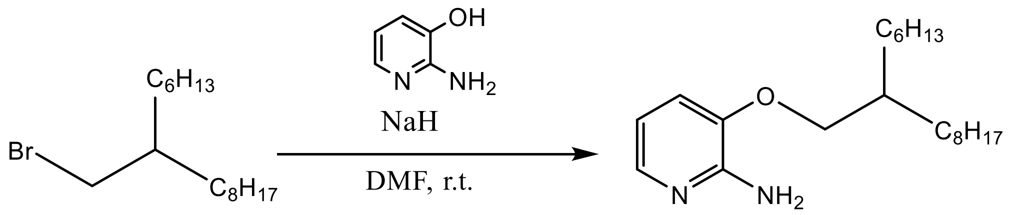

In a 500 mL flask, under N_2 atmosphere, 2-amino-3-hydroxypyridine (2.64 g, 24 mmol) and activated sodium hydride (1.40 g, 58 mmol) was stirred in a dry DMF solution (180 mL) under room temperature for 2h. And then, a solution of 7-(bromomethyl)pentadecane (10.69 g, 35 mmol) solved in dry DMF solution (10 mL) was also added. The reaction mixture stirred another 24 h in dark condition, and then was poured in extraction system of water and chloroform. The crude product was firstly purified using the column chromatography by pure hexane as an eluent to remove residual DMF, and subsequently using methanol as an eluent to obtain dark brown oil product (4.34 g, yield: 54.1 %). ^{1}H NMR ($CDCl_3$, 500 MHz, 295K): [ppm] = 7.60 (d, J = 5.3 Hz, 1H), 6.86 (d, J = 8.3 Hz, 1H), 6.56 (dd, J_1 = 7.9vHz, J_2 = 4.9 Hz, 1H), 4.75 (br, 2H), 3.82 (d, J = 5.3 Hz, 2H), 1.82-1.74 (m, 1H), 1.46-1.14 (m, 24H), 0.90-0.78 (m, 6H); ^{13}C NMR ($CDCl_3$, 125 MHz, 295K): [ppm] = 150.28, 141.99, 138.20, 115.30, 113.49, 70.84, 64.90, 53.40, 45.93, 36.85, 35.10, 32.32, 31.88, 31.82, 31.47, 29.98, 29.65, 29.56, 29.14, 22.65, 14.08.

(2) 3,6-Di(thiophen-2-yl)-2,5-dihydropyrrolo[3,4-c]pyrrole-1,4-dione

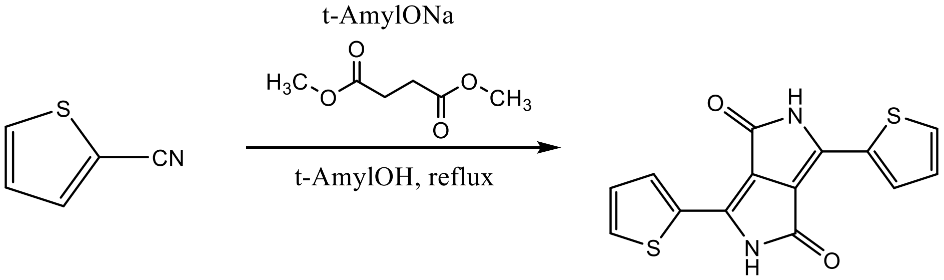

Sodium (1.1g, 48 mmol) and trace ferric chloride are mixed after weighing and added to t-amyl alcohol solution (150 ml). About 1 hour and after the sodium is completely dissolved, 2-cyanothiophene (5 ml, 53.3 mmol) was dripped slowly for 1 hour. Another 1 hour, diisopropylsuccinate (4.4 mL, 22 mmol) was added dropwise to the reaction mixture during 1.5 hours. And then the reaction mixture was refluxed about 6 h. After that, 35% hydrochloric acid and methanol were added, stirred for 0.5 hours, filtered, and washed with a large amount of water and methanol, and dried to get the dark red precipitate 4.9 g, yield about 74%., The product is used in the next step without further characterization as the result of poor solubility.

(3) PPAB

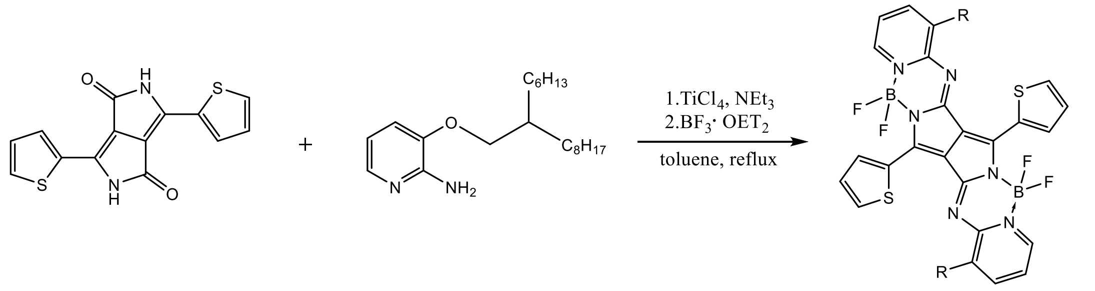

(1) (4.5 g, 13.5 mmol) and **(2)** (0.90 g, 3.0 mmol) were added to a dry toluene (180 mL), and refluxed ca.1 hour. Titanium tetrachloride (2.4 mL, 22 mmol) was added to the reaction mixture. And after 10 min, triethylamine (6.0 mL, 44 mmol) was added. After 2 hours, borontrifluoride etherate (5.7 mL, 46.5 mmol) was added, and refluxed 18 hours. Then, the reaction mixture was poured into water and extracted with dichloromethane. The combined organic extracts were dried over sodium sulfate and concentrated in vacuo to give a green solid. The crude residue was purified by silica gel column chromatography using mixed solvent (dichloromethane/hexane = 1:1

(v/v)) as the eluent to obtain the green solid 0.84 g (yield : 27%). ^{1}H NMR (500 MHz, $CDCl_3$) δ 9.39 (s, 2H), 7.89 (s, 2H), 7.64 (s, 2H), 7.21 (d, *J* = 3.9 Hz, 4H), 7.01 (s, 2H), 3.95 (d, *J* = 5.5 Hz, 4H), 2.07 (d, *J* = 12.2 Hz, 2H), 1.31 – 1.20 (m, 48H), 0.81 – 0.77 (m, 12H).

1.3 Characterization for polymers

1.3.1 Structure and molecular weight characterization

NMR spectra were obtained using a Mercury 500 spectrometer.

1.3.2 Thin film structures characterization

A 3 kW thin film X-ray diffraction (XRD) experiment was carried out on a powder X-ray diffractometer (Inca energy, Oxford Instruments). The film was prepared by drop coating of PPAB solution (5 mg / ml chloroform solution).

1.3.3 UV/vis absorption spectra of monomer and polymers

UV/vis absorption spectra were recorded using a dual-beam grating Hitachi U-4100 absorption spectrometer. The solution UV/Vis absorption spectra of PPAB were recorded in chloroform at a standard concentration (0.1 mg/ml). The thin-film UV/Vis absorption spectra were measured for a spin-coated thin film. (2 mg/ml PPAB in chloroform on a quartz glass substrate, rotary speed: 1500 r/min)

2. Figures

^{1}H NMR spectrum

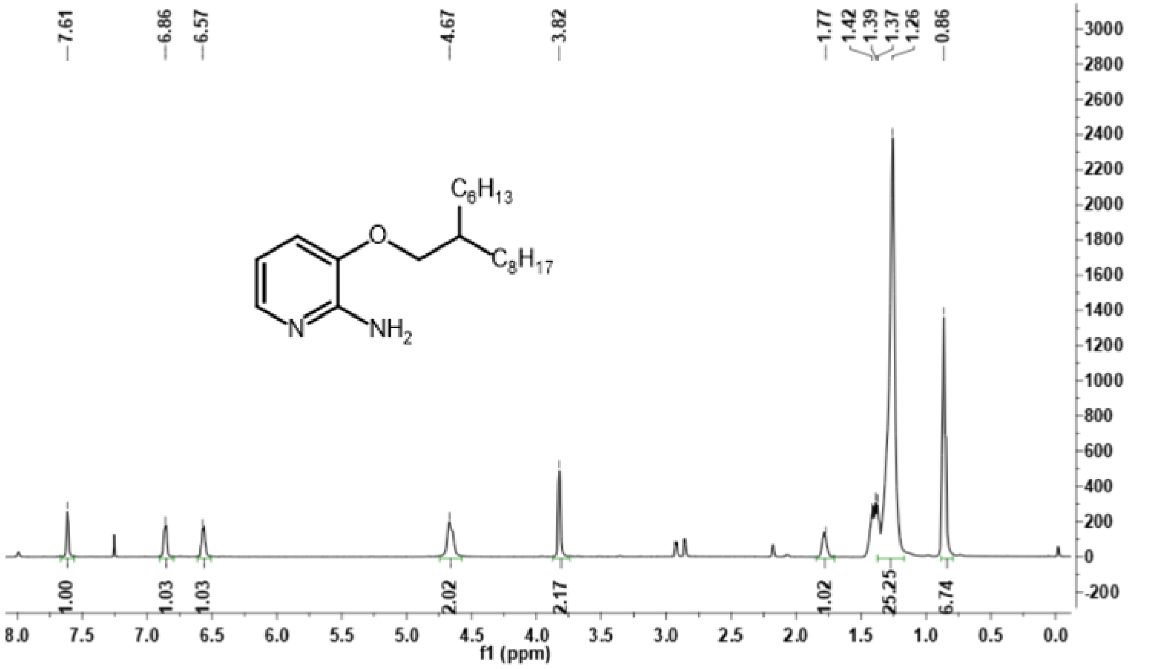

SI-Figure 1. The ^{1}H NMR spectrum of (1) 2-Amino-3-(2-hexyldecyloxy)pyridine.

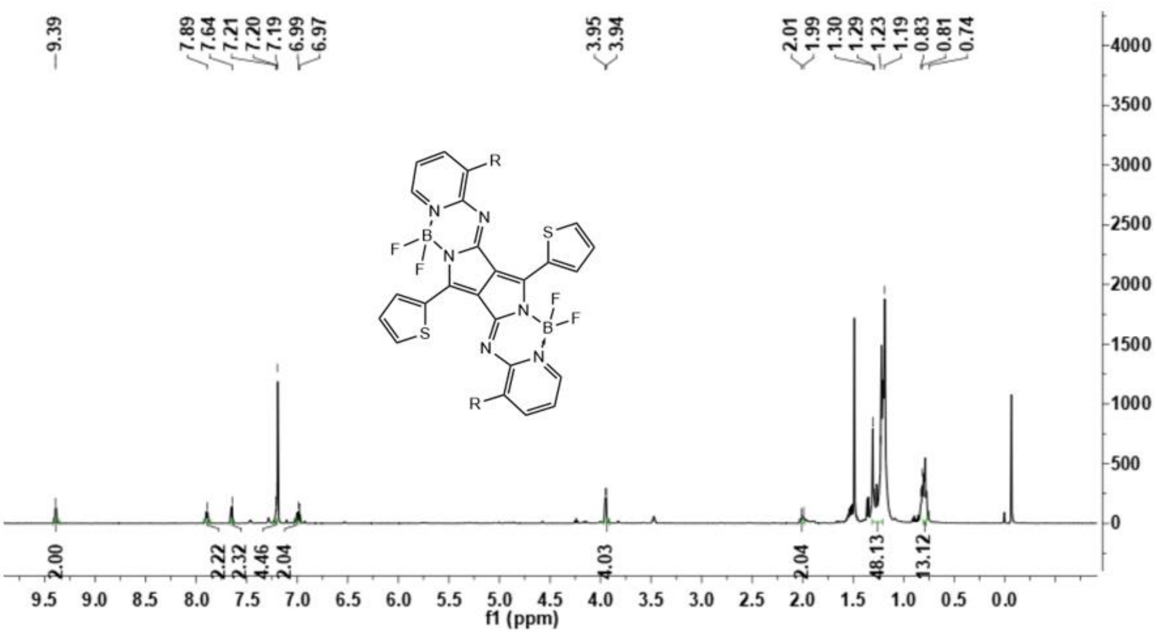

SI-Figure 2. The ^{1}H NMR spectrum of (3) PPAB.

3. Table

Table S1. The corresponding photoelectric property data of PPAB.

Sample	λ_{max} [nm]		HOMO	LUMO	E_g^{ec} / E_g^{opt}
	in solution	in thin film	[eV]	[eV]	[eV]
PPAB	692	730	-5.37	-4.16	1.21/1.37

E_g^{ec} (electrochemical band gap) according to the following equation: $-E_{LUMO} = E_{onset(red)} + 4.8$ eV and $-E_{HOMO} = E_{onset(ox)} + 4.8$ eV, where $E_{onset(red)}$ and $E_{onset(ox)}$ are the onset potentials for the oxidation and reduction processes vs. ferrocene; E_g^{opt} (optical band gap) was measured at the onset of the absorption of organic molecules film ($E_g^{opt} = 1240/\lambda_{abs.onset}$ eV)

3. Reference

[1]. Yuto Kage, Shigeki Mori, Marina Ide, et al. ***Materials Chemistry Frontiers***, **2018**, *2*, 112.

[2]. Podlesný Jan, Dokládalová Lenka, Pytela, Oldřich, et al. ***Beilstein Journal of Organic Chemistry***, **2017**, *13(1)*: 2374–2384.
